# Involvement of Mechanical Cues in the Migration of Cajal-Retzius Cells in the Marginal Zone During Neocortical Development

**DOI:** 10.3389/fcell.2022.886110

**Published:** 2022-05-16

**Authors:** Ana López-Mengual, Miriam Segura-Feliu, Raimon Sunyer, Héctor Sanz-Fraile, Jorge Otero, Francina Mesquida-Veny, Vanessa Gil, Arnau Hervera, Isidre Ferrer, Jordi Soriano, Xavier Trepat, Ramon Farré, Daniel Navajas, José Antonio del Río

**Affiliations:** ^1^ Molecular and Cellular Neurobiotechnology, Institute for Bioengineering of Catalonia (IBEC), Barcelona, Spain; ^2^ Department of Cell Biology, Physiology and Immunology, Universitat de Barcelona, Barcelona, Spain; ^3^ Network Centre of Biomedical Research of Neurodegenerative Diseases (CIBERNED), Institute of Health Carlos III, Madrid, Spain; ^4^ Institute of Neuroscience, University of Barcelona, Barcelona, Spain; ^5^ Unitat de Biofísica I Bioenginyeria, Universitat de Barcelona, Barcelona, Spain; ^6^ Centro de Investigación Biomédica en Red en Enfermedades Respiratorias, Madrid, Spain; ^7^ Senior Consultant, Bellvitge University Hospital, Hospitalet de Llobregat, Barcelona, Spain; ^8^ Department of Pathology and Experimental Therapeutics, University of Barcelona, Barcelona, Spain; ^9^ Departament de Física de La Matèria Condensada, Universitat de Barcelona, Barcelona, Spain; ^10^ University of Barcelona Institute of Complex Systems (UBICS), Barcelona, Spain; ^11^ Integrative Cell and Tissue Dynamics, Institute for Bioengineering of Catalonia (IBEC), Parc Científic de Barcelona, Barcelona, Spain; ^12^ Center for Networked Biomedical Research on Bioengineering, Biomaterials and Nanomedicine (CIBER-BBN), Madrid, Spain; ^13^ Institució Catalana de Recerca I Estudis Avançats, University of Barcelona, Barcelona, Spain; ^14^ Institut D’Investigacions Biomèdiques August Pi Sunyer, Barcelona, Spain; ^15^ Cellular and Respiratory Biomechanics, Institute for Bioengineering of Catalonia (IBEC), Barcelona, Spain

**Keywords:** Cajal-Retzius cells, cortical development, mechanical cues, marginal zone, atomic force microscopy, traction force microscopy

## Abstract

Emerging evidence points to coordinated action of chemical and mechanical cues during brain development. At early stages of neocortical development, angiogenic factors and chemokines such as CXCL12, ephrins, and semaphorins assume crucial roles in orchestrating neuronal migration and axon elongation of postmitotic neurons. Here we explore the intrinsic mechanical properties of the developing marginal zone of the pallium in the migratory pathways and brain distribution of the pioneer Cajal-Retzius cells. These neurons are generated in several proliferative regions in the developing brain (e.g., the cortical hem and the pallial subpallial boundary) and migrate tangentially in the preplate/marginal zone covering the upper portion of the developing cortex. These cells play crucial roles in correct neocortical layer formation by secreting several molecules such as Reelin. Our results indicate that the motogenic properties of Cajal-Retzius cells and their perinatal distribution in the marginal zone are modulated by both chemical and mechanical factors, by the specific mechanical properties of Cajal-Retzius cells, and by the differential stiffness of the migratory routes. Indeed, cells originating in the cortical hem display higher migratory capacities than those generated in the pallial subpallial boundary which may be involved in the differential distribution of these cells in the dorsal-lateral axis in the developing marginal zone.

## Introduction

Cajal-Retzius (CR) cells were first described by Santiago Ramón y Cajal and Gustaf Retzius (in 1890 and 1892, respectively) (Gil et al., 2014; Martinez-Cerdeno and Noctor, 2014). These cells are early-generated neurons located in cortical marginal zone/layer I that split from the embryonic preplate to form the marginal zone and the subplate when the cortical plate develops during early cortical development [(e.g., see (Soriano and Del Rio, 2005; Villar-Cervino and Marin, 2012; Kirischuk et al., 2014; Martinez-Cerdeno and Noctor, 2014; Marin-Padilla, 2015)]. Although several differences in CR cell phenotype, markers, physiology, and fate have been described in different mammals [e.g., (Cabrera-Socorro et al., 2007; Meyer, 2010; Meyer and Gonzalez-Gomez, 2018)], Reelin expressed by mouse CR cells during cortical development modulates the appropriate migration of cortical plate neurons, actively participating in neuronal network activity in developing marginal zone/layer I [e.g., (Soriano and Del Rio, 2005)]. In rodents, CR cells have the capacity to generate action potentials, establishing synaptic contacts in the marginal zone/layer I and receiving excitatory and GABAergic and non-GABAergic inputs (Frotscher et al., 2003; Soriano and Del Rio, 2005; Frotscher et al., 2009; Marin et al., 2010; Myakhar et al., 2011; Villar-Cervino and Marin, 2012; Quattrocolo and Maccaferri, 2013; Gesuita and Karayannis, 2021). Mouse CR cells are mainly generated in three neurogenic areas: the cortical hem (CH) (Takiguchi-Hayashi et al., 2004; Garcia-Moreno et al., 2007), the septum retrobulbar area (SR), and the pallial subpallial boundary (PSB) (Bielle et al., 2005). Shortly after generation, CR cells migrate through the preplate/marginal zone to populate the entire cortical surface following specific rostro-caudal and latero-tangential processes (De Carlos et al., 1995; Yamazaki et al., 2004; Bielle et al., 2005; Garcia-Moreno et al., 2007; Griveau et al., 2010; Miquelajauregui et al., 2010; Villar-Cervino et al., 2013). This dorsal-ventral migration of CR cells as well as subplate neurons thorough the preplate has been reported to play a crucial role in regionally defining the developing neocortex (Saito et al., 2019). Birthdates of cortical CR cells are between embryonic days 8.5 and 13.5 (E8.5-13.5) in the mouse, with a maximum between E9.5 and E12.5 (del Rio et al., 1995; Hevner et al., 2003; Gu et al., 2011), although a recent study points to a supply of CR cells from the olfactory bulb at protracted embryonic stages (de Frutos et al., 2016). During the first and second postnatal week, mouse CR cells disappear from layer I by programmed cell death [e.g., (Del Río et al., 1995; Del Rio et al., 1996)]. In fact, both their distribution and their differential disappearance play a role in neocortical regionalization and maturation (Ledonne et al., 2016; Riva et al., 2019).

Genetic screening of CR cells has revealed that a large number of factors are involved in their generation, migration, and maturation, such as *p73*, *p21*, *Zic1-3, Lhx5, and Fgf8*, *Tbr1, and 2*, *MDGA1*, *Emx1,* and *Emx2*, *Nectin1*, *Dmrt*, *Dbx1*, *Foxg1*, *Ebf2, Foxc1, LIM-homeobox* genes, and *miRNA9,* among others (Mallamaci et al., 1998; Hevner et al., 2001; Hevner et al., 2003; Muzio and Mallamaci, 2005; Zhao et al., 2006; Hanashima et al., 2007; Takeuchi et al., 2007; Abellan et al., 2010; Zimmer et al., 2010; Chiara et al., 2012; Zarbalis et al., 2012; Gil-Sanz et al., 2013; Hodge et al., 2013; Kikkawa et al., 2020). Concerning migration, several molecules have been identified as regulators of CR cell migration and distribution in the marginal zone, e.g., CXCL12, Eph/Ephrins, or Pax6 (Borrell and Marin, 2006; Paredes et al., 2006; Ceci et al., 2010; Villar-Cervino et al., 2013; Kaddour et al., 2020). In fact, CXCL12 (also termed Stromal Derived Factor 1, SDF-1), secreted by meningeal cells, is considered to be mainly responsible for CH-derived CR cell migration through CXCR4 and CXCR7 receptors expressed in CR cells. Surprisingly, the migration at the subpial position of CR cells in *CXCR4−/−, CXCR7−/−,* and *CXCL12−/−* mice, although affected, is largely maintained at dorsal pallial levels (Stumm et al., 2003). This is in contrast to other studies displaying relevant changes in CR cells location and cortical layering after the chemical removal of meningeal cells or genetic modification, suggesting that other factors associated with meninges are involved in CR cells migration and distribution (Super et al., 1997; Paredes et al., 2006; Dasgupta and Jeong, 2019). Angiogenic factors present in the outermost cortical blood vessels associated with meninges such as VEGF, Sema3E, and Ephrins have emerged as important cellular cues regulating the migration of CR cells, as is the case in other developmental processes [e.g., (Skaper et al., 2001; Mackenzie and Ruhrberg, 2012; Bribian et al., 2014)].

**FIGURE 1:**
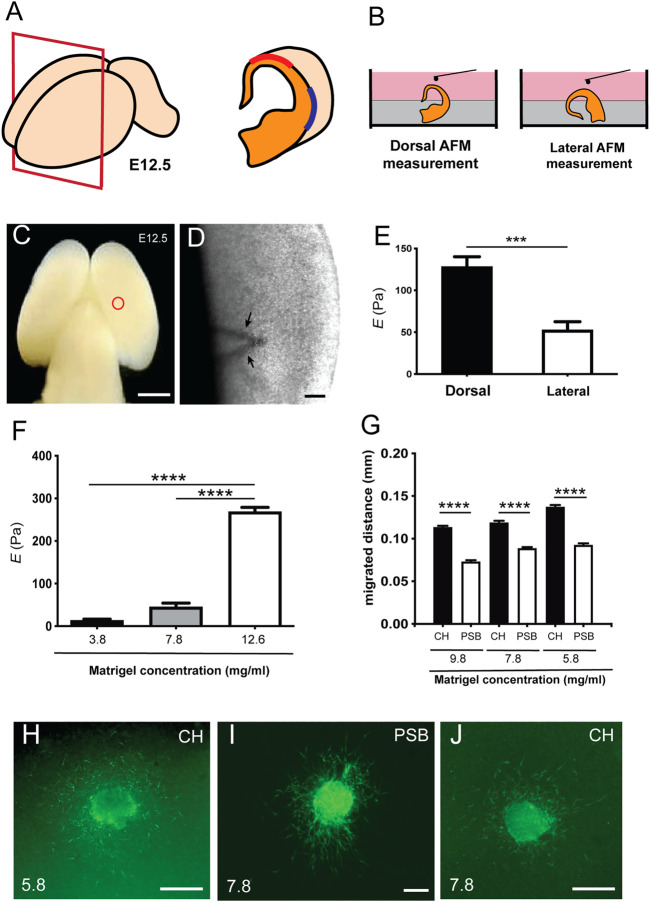
Differential stiffness between dorsal and lateral regions of the pallial marginal zone in developing mouse embryos **(A)** Scheme illustrating the procedure of placement of the telencephalic hemispheres of the embryo (E12.5). **(B)** Illustration showing the procedure for Atomic Force Microscopy (AFM), in which the whole brain embryo was first embedded in agarose to obtain dorsal and lateral measurement using the BIO-AFM. **(C,D)** Scheme **(C)** and high magnification photograph **(D)** obtained from the BIO-AFM illustrating the location of the V-shaped cantilever (circle in **C** and arrows in **D**) in the surface of the marginal zone. **(E)** Histogram showing the results of the BIO-AFM experiments; *E* values are displayed in the *y* axis in Pa. **(F)** Rheometric values obtained after the analysis of three different hydrogels. The concentration of the total protein of the analyzed hydrogels is shown in the *x* axis. **(G)** Bar plots comparing the amount of differential migration of CR cells (obtained from CH or PSB) for gradually higher Matrigel™ concentrations **(H–J)** Examples of CH **(H–J)** and PSB **(I)** cultured explants in different Matrigel™ concentrations (5.8 and 7.8 mg/ml) immunostained against CALR to identify CR cells. CH: cortical hem; PSB: pallium subpallium boundary. Data in **(E,F,G)** are presented as mean ± s.e.m.; ***p < 0.001 and ****p < 0.0001. Scale bars: C = 1 mm, D = 500 μm, H and J = 300 μm and I = 300 μm.

**FIGURE 2:**
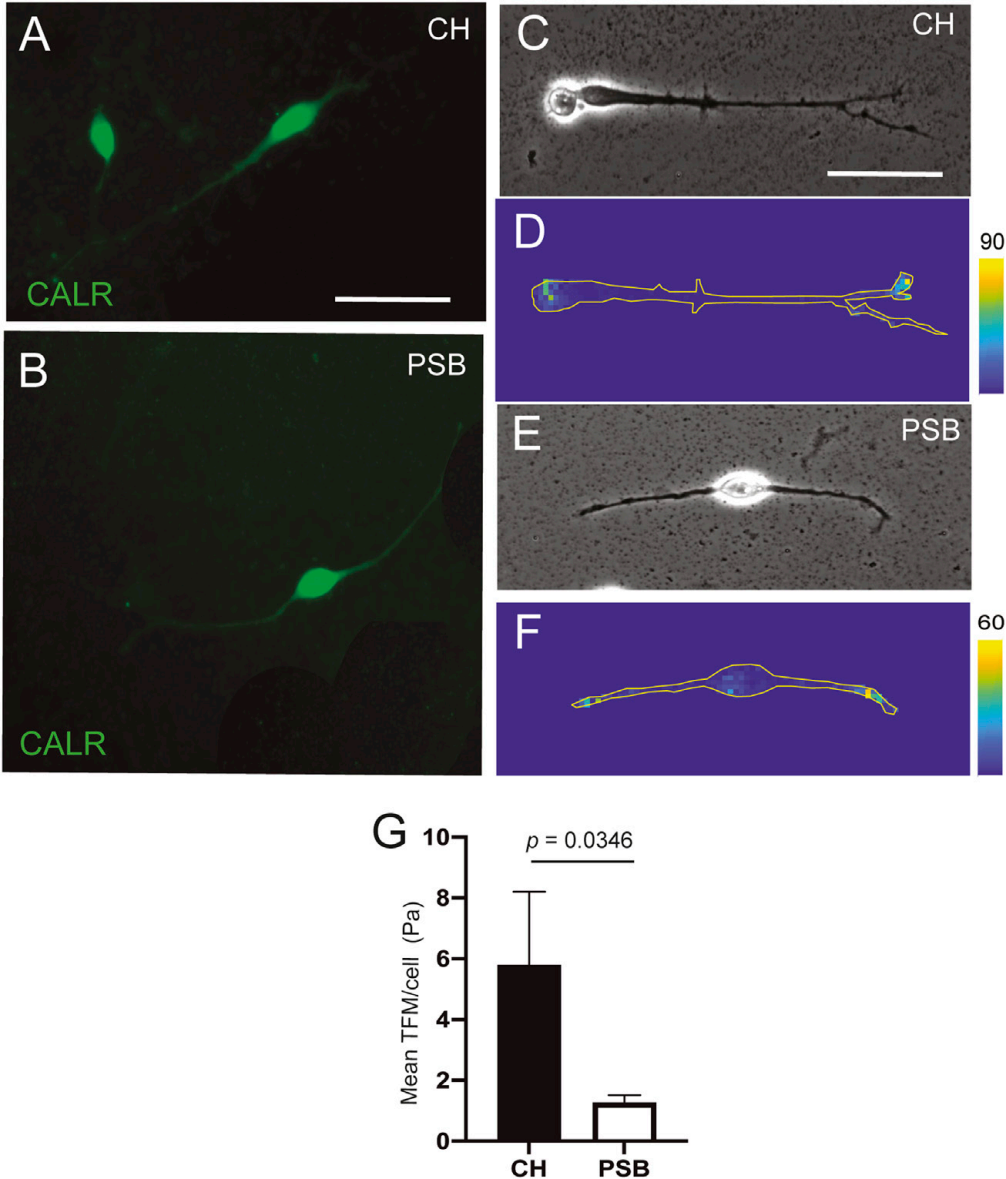
TFM measurements of CH and PSB-derived CR cells in PAA gels **(A,B)** Examples of CR cells stained using CALR antibodies derived from CH **(A)** and PSB **(B)**. **(C–F)** Phase contrast (c and e) and constraint force maps (D and F) of CR cells derived from CH **(C,D)** and PSB **(E,F)** after TFM analysis. Forces triggered by the CR cells are color-coded according to their intensity. The shape of the CR cells is outlined with a yellow contour in the constraint maps to better visualize their extent. These maps reveals that the highest traction values are morphologically located at the tips of their neurites, for both CH- and PSB-derived CR cells **(G)** Results of the Traction Force Microscopy (TFM) analysis. The bar plots show the mean pressure (force per unit area) generated by the different CR cells. The pressure values are obtained by dividing the measured forces by the total area pixels occupied by the analyzed CR cells. CH: cortical hem; PSB: pallium-subpallium boundary. Data in (g) are presented as mean ± s.e.m.. The indicated *p* value was obtained by using the one-tail permutation test. Scale bars, A = 50 μm pertains to B, C = 50 μm pertains to **(D–F)**.

In addition, evidence emerging from recent research shows that, in parallel to chemical cues, neural morphogenesis, neuronal migration, and axon navigation are processes also governed by sensing the mechanical properties of the extracellular milieu (e.g., Young’s modulus and topography) and neighboring cells during development [e.g., (Franze, 2013; Gangatharan et al., 2018; Javier-Torrent et al., 2021; Oliveri et al., 2021)]. These interactions influence the maturation and differentiation of particular neurons based on transduction of those external mechanical forces into intracellular biochemical signaling *via* a mechanical-transduction process (De Vincentiis et al., 2020; Javier-Torrent et al., 2021). This mechanical-transduction process involves the action of integrins and other elements linking extracellular matrix (ECM) to cellular cytoskeleton dynamics [see (Elosegui-Artola et al., 2018) for review]. In addition, specific signaling mechanisms such as mechanosensory receptors (e.g., Piezo1) and Hippo/YAP pathways are players in the mechanical transduction process in several cell types and tissues [e.g., (Nguyen-Lefebvre et al., 2021; Wu and Guan, 2021)], including neural tissue [e.g., (Sahu and Mondal, 2021)]. Considering matrix stiffness during mouse cortical development, Iwasita and coworkers measured, by means of Atomic Force Microscopy (AFM), the values for Young’s/Elastic modulus (*E*) of the cortical plate (CP), the intermediate zone (IZ), the subventricular zone (SVZ), and the ventricular zone (VZ) in coronal sections of the prospective parietal cortex of the mouse at different postnatal stages (from E12.5 to E18.5) (Iwashita et al., 2014). In a broad sense, all values (for all cortical layers) increased from E12.5 with a peak at E16.5 and then decreasing (see Figures 1, 2 in (Iwashita et al., 2014)) at late (E18.5) embryonic stages. Young’s modulus values ranked (for the CP) from 30.1 Pa at E12.5 to a maximum of 108.4 Pa (E16.5), decreasing to 57.4 Pa at E18.5. However, preplate and their derivatives: the molecular layer/layer I were not thoroughly analyzed in the study.

Concerning CR cell migration, a pioneer study described differences in the migratory properties of these cells depending on their origin (rostral *vs*. medial) after ectopic transplantation in different areas of the embryonic cortical hem (Ceci et al., 2010). Thus, the study revealed, for the first time, that the local environment in parallel to guidance molecules can modulate the migration of CH-derived CR cells (Ceci et al., 2010). In addition, Barber et al. (2015) *.* demonstrated differences in migratory speed of subsets of CR cells. In fact, using *in vitro* experiments of the complete pallium, Barber et al. (2015). described how SR-derived and CH-derived CR cells migrate and then stop their migration at the dorsal pallium levels; whereas PSB derived CR cells migrate in the rostral caudal axis in the lateral part of the pallium (Barber et al., 2015).

In this study, we aimed to explore whether putative differences in the mechanical properties between dorsal and lateral parts of the developing marginal zone/layer I also influenced the migration and distribution of CR cells derived from the CH and the PSB of the mouse. Our data reveal that both the stiffness differences between medial and lateral regions of the pallial marginal zone as well as the intrinsic mechanical properties of CR cells contribute to their migration from the CH and PSB in the dorsal and lateral parts of the developing neocortex.

## Material and Methods

### Animals

The following mice and rat strains were used in the present study: OF1 mice (E12.5) (RRID: MGI:5649743) and Sprague Dawley rats (E14.5) (RRID: MGI:5651135) were purchased from Charles River laboratories (Paris, France). In addition, the mTmG mice (ROSA^mT/mG^; RRID: IMSR_JAX:007576; The Jackson Laboratories; Bar Harbor, ME, United States) were also used. *Plxnd1-eGFP* mice were obtained from the Mutant Mouse Regional Resource Center (MMRRC; RRID: MMRRC_015415-UCD; University of California, CA, United States). mTmG and *Plxnd1-eGFP* mouse genotypes were verified by observing a tail fragment under a fluorescence microscope (BX61; Olympus Corporation). In addition, CXCR4-eGFP transgenic mice (RRID: MMRRC_015859-UCD; kindly provided by J.H.R. Lubke [Germany) (Anstotz et al., 2014)] were used. All the animals were kept in the animal facility of the Faculty of Pharmacy at the University of Barcelona under controlled environmental conditions and were provided food and drink ad libitum. For AFM experiments, OF1 pregnant mice were housed in the animal facility of the Faculty of Medicine at the University of Barcelona. All the experiments were carried out following protocols of the Ethics Committee for Animal Experimentation (CEEA) of the University of Barcelona (OB47/19, C-007, 276/16, and 47/20).

### CH and Pallial Subpallial Boundary Explant Dissection

In order to obtain CH and PSB regions, embryonic brains (E12.5) were covered with a mixture of L15 medium (31415-029, Invitrogen) containing 4% low melting point agarose (50111, Lonza) which was then allowed to solidify at 4°C for a few minutes. After gelation, 300 µm-thick coronal sections were obtained using a vibratome (VT1000S, Leica). Free-floating slices were collected in cold 0.1 M PBS containing 0.6% glucose and selected under a dissection microscope, and the explants of CH or PSB were dissected.

### Culture of CH and Pallial Subpallial Boundary-Derived Explants on Hydrogels

For explants embedded in hydrogels, a sandwich procedure was used using a base of homemade rat tail type I collagen (Gil and del Rio, 2012) and a top of Matrigel™ (354434, Corning, Cultek). To prepare the Matrigel™ at different densities, cold Neurobasal™ media (21103-049, Invitrogen) was used to dilute Matrigel™ stock solution. One explant per preparation was placed on a homogeneous collagen base and then another layer of Matrigel™ was added on top. Before the Matrigel™ gels, explant position was verified and correctly positioned under the dissecting microscope. Once the explant was seeded, the gel was allowed to coagulate at 37°C, 5% CO_2_ before adding the supplemented medium. At 2 days *in vitro* (DIV), explants were fixed for 1 h with cold 4% buffered paraformaldehyde (PFA) before washing with 0.1 M PBS, and then stored at 4°C prior to immunocytochemistry or photodocumentation. The number of cells that migrated out of the explants was counted, and the maximum distance migrated from the cell body to the explant edge was also determined using Fiji™ software, using as calibration pictures of a millimetric eyepiece at the same magnification. In some experiments, CH-derived explants were treated with 4 μg/ml of Cytochalasin D (C8273, Sigma-Aldrich), 10 μM Nocodazole (M1404, Sigma-Aldrich), or 0.5 μg/ml of Blebbistatin (1760, Tocris).

### *In vitro* Transplantation of CH or Pallial Subpallial Boundary Explants in Telencephalic Slices

Brain slices were obtained from E12.5 wild-type mice and the CH and the PSB from E12.5 mTmG mouse embryos as described [see also (Bribian et al., 2014)]. Slices were transferred to collagen-coated culture membrane (PICM0RG50, Millipore) in 1.2 ml of medium BME-F12 1:1 (41010-026, Invitrogen), glutamine (25030-024, Invitrogen), 5% horse serum (26050-088; Invitrogen), penicillin, streptomycin (15140122, Invitrogen), and 5% bovine calf serum (12133C, Sigma-Aldrich). The CH and PSB from wild-type slices were removed and replaced with the dissected CH or the PSB from mTmG mice slices. After several hours, the medium was changed to Neurobasal™ medium supplemented as above and cultured for up to 48 h before analysis.

### *In situ* Hybridization

*In situ* hybridization was carried out as described previously (Mingorance et al., 2004; Mata et al., 2018) on 50 μm vibratome fixed brain sections of E12.5 embryos. Both sense and antisense riboprobes against *Wnt2b* (provided by P. Bovolenta) and *Reln* (provided by T. Curran) were labelled with digoxigenin, according to the manufacturer’s instructions (Roche Farma).

### Immunocytochemical Methods

The processing of each *in vitro* model was determined by its culture characteristics (hydrogel, coverslip, or brain slice). Although the general procedure was similar for all conditions, the incubation times and mounting methods for analysis differed. The general procedure started with fixing the tissue samples with 4% PFA, then washing them with 0.1 M PBS and a blocking solution composed of 10% Fetal Bovine Serum (FBS; 10500064, Invitrogen) and 0.1 M PBS with Triton X-100 (Sigma-Aldrich) (concentration determined by each model). After washing with PBS-Triton X-100, the primary antibody was incubated with 7% FBS and PBS−0.2% gelatin and Triton X-100. This was followed by the Alexa-tagged secondary antibodies (Alexa 488; A21206, Invitrogen) diluted in 7% serum (FBS) and PBS 0.1 M containing gelatin 0.2% and 0.1% Triton X-100. Afterwards, nuclear staining was performed using Hoechst (1 μg/ml; B2261, Sigma-Aldrich). The antibody used for CR cell labeling was Calretinin (CALR; 1:1,000; 7,697, Swant Antibodies). Details of the immunocytochemical procedures in each model are briefly explained below. For explant cultures growing in Matrigel™ 0.5% Triton X-100 was used in all steps and long incubation times were observed. After fixation for 1 h at 4°C with 4% buffered PFA and blockade for 4 h at room temperature, the primary antibody was incubated for 2 overnights at 4°C and the secondary antibody for 1 overnight at 4°C with gentle shaking. Finally, nuclear labeling with Hoechst was developed for 20 min before washing with 0.1 M PBS and mounting with Mowiol™ (475904, Calbiochem). For primary cultures of CH or PSB-derived CR cells growing on polyacrylamide (PAA) gels for Traction Force Microscopy (TFM) experiments, a short fixation time (5 min) with 2% buffered PFA (removing half of the medium), and 10 min in 4% buffered PFA at 4°C, was developed in selected preparations. Thereafter, fixed neurons were incubated at room temperature for 1 h with blocking solution, 2 h with primary antibodies, and 1 h for the secondary antibodies, at room temperature with gentle shaking. Hoechst staining was run for 10 min before washing with 0.1 M PBS after immunohistochemistry and photodocumentation. For slice cultures, coronal slices after CH or PSB transplantation (see above) were fixed for 1 h with 4% buffered PFA, and after this detached from the transwell membrane and free-floating processed with gentle agitation. All the immunohistological solutions contained 0.5% Triton X-100 and the samples were incubated for longer times. Thus, slices were treated with blocking solution for 4 h, incubated with primary antibodies for 48 h, and then for 12 h with secondary antibodies at 4°C with gentle shaking. Finally, the Hoechst solution was incubated for 20 min before washing with 0.1 M PBS and mounting with Mowiol™; double labelled CALR-mTmG-positive CR cells were photodocumented using a Zeiss SLM800 confocal microscopy.

### Viscoelastic Properties of Matrigel™ Hydrogels Analysed With Rheometry

The different densities checked in the study were obtained from a Matrigel™ stock with known total protein concentration diluted in cold Neurobasal™ medium. The dilutions were different for different batches of Matrigel™: 12.72 mg/ml (Lot nº: 9294006), 12.6 mg/ml (Lot nº: 8015325), 11.95 mg/ml (Lot nº: 9021221), and 9.8 mg/ml (Lot nº: 9148009). To obtain the hydrogels, a minimum of 100 µl of mixture was needed per dish, carefully depositing the mixture and leaving it to gel for 2–4 h at 37°C. Once gelled, complete Neurobasal™ medium was added to cover, and left in an incubator at 37°C and 5% CO_2._ At 2 DIV the dishes were carefully lifted and placed on a rheometer plate previously heated to 37°C and calibrated. For rheometry measurement of the hydrogels, an ∅ 8 mm Peltier (Peltier plate Steel — 108990) coupled to a Discovery Hybrid Rheometer HR-2 (Discovery HR-2; 5,332-0316; TA instruments) was used. To prevent evaporation, rapeseed oil was used as a solvent-trap after applying loading gap to the sample and deleting medium and excess hydrogel. Finally, the geometry was taken to the gap to geometry and the chosen measurement began. With the TRIOS program (v. 5.0.0.44608) the Frequency sweep test using a gap of 0.5 mm (previously determined) was selected. To obtain the elastic modulus *E*, we first measured the storage and loss moduli in experiments at 1 Hz frequency sweep, which provided a strain modulus *G* given by the equation:

$$G=\sqrt{{\left(G^{\prime }\right)}^{2}+{\left(G^{\prime \prime }\right)}^{2}}$$

Where *G′* corresponds to storage modulus and *G″* to loss modulus. Once *G* was obtained, the elastic modulus *E* values were determined as equation:

E=2G(1+υ)

Where υ is the Poisson’s ratio, defined as the ratio of transverse contraction strain to longitudinal extension strain, and that was assumed to be 0.5 for low stiffness hydrogels.

### Atomic Force Microscopy Experiments

For *in situ* AFM measurements, whole E12.5 mouse embryonic brains were carefully dissected without damaging the cortical surface. Then, a 4% agarose (SeaPlaque™ GTGTM Agarose, 50111; Lonza) solution was prepared in 0.1M PBS and left at 45°C in a dry bath. The whole brain was dissected and examined at dorsal and lateral regions of the pallium for AFM analysis (Figure 1). Two brain orientations were generated in embedding the whole brain in specific orientation with agarose to achieve dorsal and lateral AFM measurements of the brain surface avoiding the supallial areas (Figure 1B). Briefly, a large plate, with a glass slide for AFM calibration, containing 2–3 mm thickness of 4% agarose, was prepared. Once jellified, one hemisphere was carefully placed over the agarose surface adding more agarose to embed the subpallial brain regions, leaving the dorsal pallial area to develop the dorsal AFM measurements (Figure 1B). In parallel experiments, the other whole hemisphere was placed laterally over the bottom agarose with the medial brain portion in contact with agarose, being leaving the lateral part of the pallium of the agarose-embedded brain for AFM measurement (Figure 1B). After gelling, the plate was covered with complete Neurobasal™ medium and placed in the incubation chamber at 37°C.

Measurements were carried out on a custom-made BIO-AFM mounted on an inverted optical microscope (TE 2000; Nikon). AFM was equipped with a V-shaped silicon nitride cantilever (0.01 N/m nominal spring constant) terminating in a 6 μm-radius borosilicate spherical tip (Novascan Technologies). The cantilever deflection was measured by using the optical lever method, and the sensitivity of the photodiode was calibrated prior to probing each sample by using the agarose semi-embedded glass slide in the preparation as reference. For each measurement (dorsal or lateral), 4 separate probing points were selected by laterally displacing the AFM probe 40 µm between measurements. For each probing point, the *E* modulus was calculated from the force-displacement curves by adjusting the Hertz model for the tip-surface contact (Alcaraz et al., 2018). From these 4 separate values, the average was calculated, and data were represented by mean ± standard error of the mean (s.e.m.) for each brain at the different positions.

### Primary Cultures and Traction Force Microscopy Measurements of CH or Pallial Subpallial Boundary-Derived CR Cells

Pieces of CH and PSB were obtained as above and collected in cold dissection media (0.1M PBS (14200, Invitrogen) containing 0.65% glucose (G8769, Sigma-Aldrich)) and centrifuged for 5 min at 800 rpm. After removal of the dissection media, 3 ml of dissection medium containing 10X trypsin (15400-054, Invitrogen) at 37°C for 15 min was added. After digestion and inactivation with heat-inactivated normal horse serum (1:3 ratio); 10X DNase (AM2222, Ambion) diluted in fresh dissection media was added, and incubated for 15 min at 37°C. Finally, 10 ml of dissection medium was added and centrifuged for 5 min at 800 rpm. The pellet was resuspended in complete Neurobasal™ medium, and the cells were seeded on the plate. Then cell density was approximately 100,000 cells per 9.5 cm^2^ plate. For acrylamide gels, a Matrigel™ coating (diluted 1:40) was made the day before and incubated at 37°C overnight. The next day, the surface was rinsed with Neurobasal™ medium. For the TFM assay, only isolated cells with clear morphology of CR cells (see below) were analysed to avoid interference from traction forces between different cells. PAA gels with different stiffness were generated by modifying the proportion of acrylamide 40% (1610140; BioRad) and Bis-acrylamide Solution (2% w/v; 10193523; ThermoFisher) and the level of crosslinking in the gel (between ≈40 Pa up to supraphysiological values) (Nocentini et al., 2012). To detect gel displacements due to CR cell mediated forces, the PAA gels was labelled with FluoSpheres^®^ Carboxylate-Modified Microspheres 0.2 µm [(625/645) F8806; LifeTechnologies]. In order to generate a good homogeneous Matrigel™ coating for CR cells, the PAA gels were treated with SulfoSANPAH (803332; Sigma-Aldrich). Thus, 0.1 M PBS was removed and a mixture of SulfoSANPAH (and 20 µl of reagent diluted in 480 µl bidistilled water) was added. After two rinses with 0.1M PBS, treated gels were coated overnight at 37°C. The following day, coated gels were washed with the fresh culture medium and allowed to stabilize for a few minutes before seeding with CR cells. Once adhered to the PAA gel, isolated CR cells were selected ( × 40 objective, inverted Olympus microscope I×71). For image acquisition and data processing, a Matlab™ script was used (see (Reginensi et al., 2015) for details), and the displacements were represented on the bright field image of the cell.

### Calcium Analysis in Cultured CR Cells With Fluo4-AM

To develop analysis of the changes in Ca^2+^ levels in CR cells, CH-derived explants were cultured on Matrigel™ coated dishes as indicated above. In order to enhance explant adhesion and correct CR migration, culture media contained 1% methylcellulose. In previous experiments, we determined the appropriate concentration of methylcellulose maintaining the morphology of CALR-positive cells (Supplementary Figure S1). After 24 h of culturing, explants were incubated for 30 min with the cell–permeant calcium-sensitive dye Fluo4-AM (F14201, Molecular Probes). The culture was washed with fresh medium after incubation and finally placed in a recording chamber for observation. The recording chamber was mounted on an IX71 Olympus inverted microscope equipped with a Hamamatsu Orca Flash 4.0 CMOS camera (Hamamatsu Photonics). Cultures were recorded and images (1,024 × 1,024 pixels) were captured using a 20× objective and 470 nm wavelength (CoolLED’s pE-300^white^, Delta Optics) every 50 ms for 1 min using the CellSens™ software (Olympus). The recordings were analyzed offline using the Matlab™ toolbox NETCAL (www.itsnetcal.com). Identified CR cells were associated with a single region of interest (ROI). The average fluorescence *Fi (t)* in each ROI (CR cell) *i* along the recording was then extracted, corrected for global drifts and artifacts, and finally normalized as *(Fi (t) — F*_*(0,i)*_ *) / F*_*(0,i)*_ *= fi (t)*, where *F*_*0,i*_ is the background fluorescence of the ROI. The time series of *fi (t)* was analyzed with NETCAL to determine sharp calcium transients and that reveal neuronal activity. Obtained movies were edited in Fiji™ and the lockup table “physics” was applied. In this experiment, the mechanosensory channel inhibitor GsMTx-4 (ab141871, Abcam) was used at a final concentration of 10 μg/ml during video recording.

### Statistical Analysis

Data in this manuscript are expressed as mean ± s.e.m. of at least four independent experiments unless specified. Means were compared using the Mann-Whitney *U* non-parametric test. The asterisks ∗∗, ∗∗∗ and ∗∗∗∗ indicate *p* < 0.01, *p* < 0.001 and *p* < 0.0001, respectively. For TFM and AFM analysis, a permutation test (one tail) was performed and ∗, ∗∗ indicate *p* < 0.05 and *p* < 0.01, respectively was considered statistically significant. Statistical test and graphical representation were performed with Prism v.8 (GraphPad Software), RStudio (RStudio, PBC), and R software (The R Foundation).

## Results

### Dorsal to Lateral Stiffness (*E*) Differences are Present in Developing Marginal Zone of Developing Mouse Cortex

As a first set of experiments, we developed BIO-AFM measurements in dorsal and lateral parts of the pallial surface of embryonic mice (E12.5) (Figure 1). At this embryonic stage, a lateral growth of the pallium takes place (Jacobson and Rao, 2005) and CR cells tangentially migrate through the marginal zone and cover the entire pallial surface (see introduction for references). In our experiments we focused on the dorsal and lateral portions of the pallium, avoiding the most ventral/subpallial regions of the telencephalon. By placing the brain semi-embedded on agarose, we can immobilize the brain without disrupting the whole preparation and leaving a free-agarose zone for the BIO-AFM measurements (Figure 1B). A representation of the dorsal zone is showed in low magnification and at higher magnification the cantilever can be seen across the brain tissue (Figures 1C,D). Our BIO-AFM results indicate clear differences in stiffness between the dorsal and lateral portions of the pallial surface (dorsal: 128.1 ± 12.08 Pa *vs*. lateral: 52.46 ± 12.08 Pa, mean ± s.e.m., ∗∗∗*p* = 0.0002) (Figure 1E) (*n* = 9 for each condition).

### Differential Migration of CH and PSB-Derived CR Cells in Different Matrigel™ Concentrations

Next, we aimed to explore whether these stiffness differences might affect the migration of CR cells (Figures 1F–J). Classical studies analyzing CR cell migration used 3D-Matrigel™ hydrogels as a migration substrate [e.g., (Borrell and Marin, 2006; Bribian et al., 2014)]. In order to determine whether the stiffness of the environment could modulate the migration of CR cells in a region-specific manner, we first analyzed the stiffness of different Matrigel™ concentrations using rheometric analysis (Figure 1F). We selected the Matrigel™ concentrations taking into account the total protein level in the different batches obtained from the supplier (see Materials and Methods for details) in terms of the generation of a homogenous hydrogel at each of these concentrations [see (Gil and Del Rio, 2019) for example]. Data illustrate that, as expected, high Matrigel™ concentrations led to significantly higher Elastic moduli *E* (Figure 1F), with *E* decreasing from 12.6 mg/ml to 3.8 mg/ml Matrigel™ dilutions. Measured *E* at 1 Hz for 12.6 mg/ml was 268 ± 10.11 Pa (*n* = 6), for 7.8 mg/ml it was 45.39 ± 8.93 Pa (*n* = 5), and for 3.8 mg/ml it was 13.34 ± 3.02 Pa (*n* = 4), data as mean ± s.e.m. (Figure 1F). When comparing the elasticity of the hydrogels with those previously obtained in BIO-AFM, *E* values obtained using 12.6 mg/ml of Matrigel™ were around 2 times than those measured in the *in vivo* BIO-AFM. However, the data obtained using 7.8 mg/ml were similar to those observed in lateral regions of the apical surface and the values using 3.8 mg/ml were 4 times lower than the lateral telencephalic portion of the marginal zone. For this reason, we used three different concentrations of Matrigel™ ranging around physiological values as measured: 5.8 mg/ml, 7.8 mg/ml, and 9.8 mg/ml.

As indicated in several studies, CR cells derived from the CH and PSB showed an overlapping distribution in the developing marginal zone-layer I [e.g., (Barber and Pierani, 2016)]. Taking this into account, we cultured CH and PSB-derived explants in Matrigel™ hydrogels with different concentrations. CH-derived CR cells were able to migrate longer distances in 5.8 mg/ml, 7.8 mg/ml, and 9.8 mg/ml Matrigel™ dilutions (values for 9.8 mg/ml: CH = 113.4 ± 1.7 μm (*n* = 1632) *vs*. PSB = 72.86 ± 1.3 μm (*n* = 915). Values for 7.8 mg/ml: CH = 131.7 ± 2.1 μm (*n* = 1471) *vs*. PSB = 88.52 ± 1.4 μm (*n* = 1911). Values for 5.8 mg/ml: CH = 137.1 ± 2.2 μm (*n* = 1737) *vs*. PSB = 92.35 ± 2.2 μm (*n* = 1194); all mean ± s.e.m.) (Figure 1G; Supplementary Materials movies S1, S2). This suggests that 1) CH-derived CR cells have greater motogenic capacity than PSB-derived CR cells when migrating in hydrogels with the same *E* value, and 2) CH-derived CR cells are able to migrate greater distances in hydrogels displaying *E* values closer to those observed in the dorsal portion of the pallium in contrast to PSB-derived CR cells. In Figures 1H–J we offer some examples of the distribution of CALR-positive CR cells after completing their migration for 2 DIV in different Matrigel™ concentrations.

### Hem-Derived CR Cells Displayed Greater Mechanical Forces Than PSB-Derived CR Cells in PAA Gels

**FIGURE 3:**
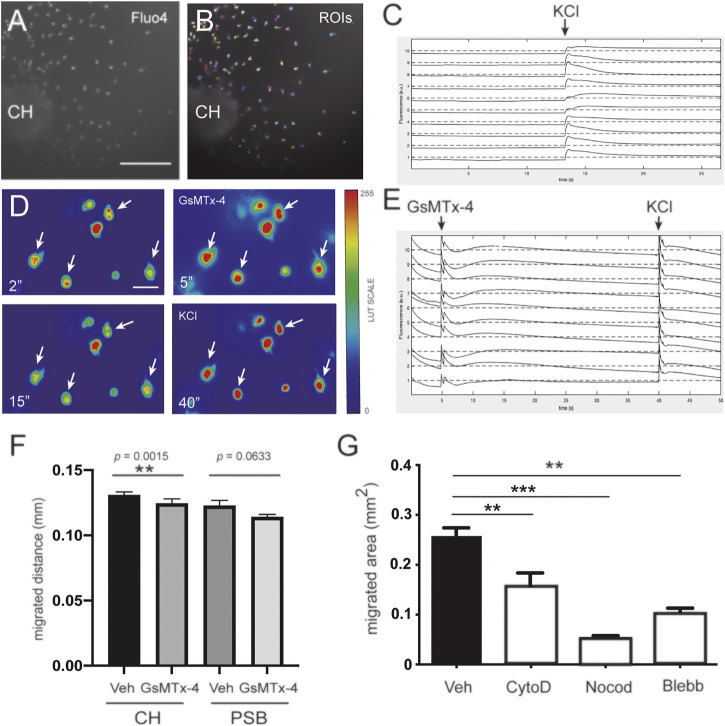
Involvement of mechanosensory receptors in the migration of CH-derived CR cells **(A–C)** Fluorescent calcium imaging experiments demonstrating that CR cells are able to depolarize in the presence of KCl. The images in panel A corresponds to representative neuronal cultures of CH on methylcellulose-containing medium **(A)**. The same image is shown in panel B together with the detected regions of interest (ROIs, colored patches) and that ascribed as neuron **(B)**. **(C)** The application of 0.1 M KCl is indicated with an arrow. The abrupt increase in fluorescence reveals neuronal response to the chemical stimulation. **(D)** Fluorescence images at preset time points (2, 5, 15, and 40 s) from identified CR cells illustrating the changes in Ca^2+^ upon application of GsMTx4 (at 5 s) and KCl (at 40 s). Images were extracted from the Supplementary Material Movie S3). Fluorescence images were color edited to visually enhance the transient decrease in Ca^2+^ after GsMTx4 (at 5 s) and KCl (at 40 s). White arrows help identifying the fluorescence evolution of four neurons **(E)** Fluorescent traces for 10 representative neurons upon treatment with GsMTx4 (at 5 s) and KCl (at 40 s), highlighting their strong response to stimulation **(F)** Bar plots comparing the effect of GsMTx4 treatment on the migration of CH- and PSB-derived CR cells, and relative to untreated, control cells. CR cells exhibit a larger migration distance as compared to PSB ones **(G)** Bar plots comparing the migration capacity of control CR cells with those treated with cell-mobility blockers, namely cytochalasin D, Nocodazole, and Blebbistatin. For panels **(F,G)**, data are presented as mean ± s.e.m.. Veh: Vehicle; CytoD: Cytochalasin D; Noco: Nocodazole; Bleb: Blebbistatin; CH: cortical hem; PSB: pallium subpallium boundary. The specific *p* values are included in **(F)**, and **p < 0.01 and ***p < 0.001 in **(F,G)**, respectively. Scale bar A = 300 μm pertains to B; D = 50 μm.

In a next set of experiments, we aimed to determine whether these migratory differences could be attributed to intrinsic differences between CH- or PSB-derived CR cells in order to generate mechanical forces when cultured on PAA substrates. First, we generated PAA gels with very low *E* using the protocol published in (Sunyer et al., 2012; Sunyer et al., 2016). We obtained soft PAA gels with *E* values around **≈** 40–100 Pa. This *E* value is the lowest stiffness that allowed us to obtain good distribution of the nanoparticles used in TFM. Below these values the PAA is not stable and does not generate reliable TFM measurements. We aimed to analyze the behavior of CH- and PSB-derived CR cells when cultured on these low-Pa PAA gels. CR cells adhered to the PAA gel and did not migrate but generated forces on the substrate (Figure 2). In some cases, due to the absence of a 3D-hydrogel environment, CR cells modify their morphology from the typical unipolar to a more bipolar shape as also observed in other studies in 2D-cultures (Villar-Cervino et al., 2013). In previous experiments we also categorized the cell morphologies as CR cells in PAA gels using CALR immunostaining (Figures 2A,B). Thus, we developed the TFM measures in isolated CR cells with these morphologies (Figures 2C, F) and did not analyze TFM in cells with multipolar morphology nor did we group them to compare equal populations. TFM results demonstrated, as expected, that CR cells independently of their origin, do not generate large forces to the PAA substrates compared to other cell types [e.g., endothelial cells or fibroblasts, (Roca-Cusachs et al., 2017)]. TFM analysis reported that CH-derived CR cells can develop greater forces on the substrate when compared to PSB-derived ones (*p* = 0.0346, one tail permutation test, *n* = 18 and 14, respectively) (Figure 2G). This also points to differing intrinsic mechanical properties between CH and PSB-derived CR cells that might allow CR cells to sense the different stiffness of the marginal zone. One potential mechanism to sense stiffness is the expression of mechanosensory channels (Roca-Cusachs et al., 2017). To assess this, we developed a loss of function experiment using the mechanosensory channel inhibitor GsMTx-4 (Gnanasambandam et al., 2017) (Figure 3). This compound is a spider venom that inhibits cationic mechanosensitive channels. In fact, although the specific mechanisms of the drug have not been fully determined, when GsMTx-4 is applied to several cell types expressing mechanosensitive channels (e.g., Piezo channels) Ca^2+^ influx is blocked (Jacques-Fricke et al., 2006). Taking this into account, we cultured hem-derived explants, and after 40 h in order to obtain isolated CR cells, cultures were incubated with Fluo4-AM. After incubation, the changes in the Ca^2+^ waves in CR cells were analyzed using NETCAL Software (Orlandi et al., 2014). First, we checked the health status of cultured CR cells in 1% methylcellulose containing medium by analyzing their depolarization, using KCl (Figures 3A–C). After KCl treatment, an increase in the fluorescence Δ*F/F0* values was observed in all analyzed CR cells (Figure 3C). Next, we developed similar experiments, first incubating CR cells with the inhibitor GsMTx-4 and then after that with KCl (Figure 3D–E). Results demonstrated that treatment with GsMTx-4 transiently decrease intracellular calcium levels in CH-derived CR cells, reducing their migration (CH, Veh = 130, 8 ± 2.5; GsMTx-4 = 124, 4 ± 3.5, mean ± s.e.m., ∗∗*p* = 0.0015, *n* = 1,218 and 782, respectively) (Figures 3E,F). On the graph (Figure 3E) there is a first fluorescence increase by GsMTx-4 application by medium disruption, but after that, the cell senses the inhibitor and react with a second peak. As a result of the inhibitor entry, the cell decreases their calcium activity and decrease their levels under the baseline. For PSB, CR-cell migration was lower after incubation with the inhibitor but did not reach statistical significance (*p* = 0.063, *n* = 1,289 for GsMTx-4, and *n* = 382 for vehicle) (Figure 3F). In addition, we analyzed whether the blockage of cytoskeletal proteins and myosin II also impaired their migration. These experiments showed, as expected, that inhibiting tubulin (Nocodazole; ∗∗∗*p* < 0.001, *n* = 21) and myosin II (Blebbistatin; ∗∗*p* = 0.0032, *n* = 22) almost blocked the migration of CR cells (Figure 3G). In addition, when inhibiting actin dynamics, we also achieved an inhibition of CH-derived CR cell migration (Cytochalasin D, 4 μg/ml; ∗∗*p* = 0.0045, *n* = 9; Figure 3G). Taken together, the present data demonstrate that CR cells can generate mechanical forces to the substrate (CH > PSB-derived CR cells), but that cytoskeletal disruption impairs their migration on Matrigel™ hydrogels.

### Ectopic Transplantation of CH and PSB-Derived CR Cells Demonstrates Intrinsic Mechanical Properties *in Vitro*.

**FIGURE 4:**
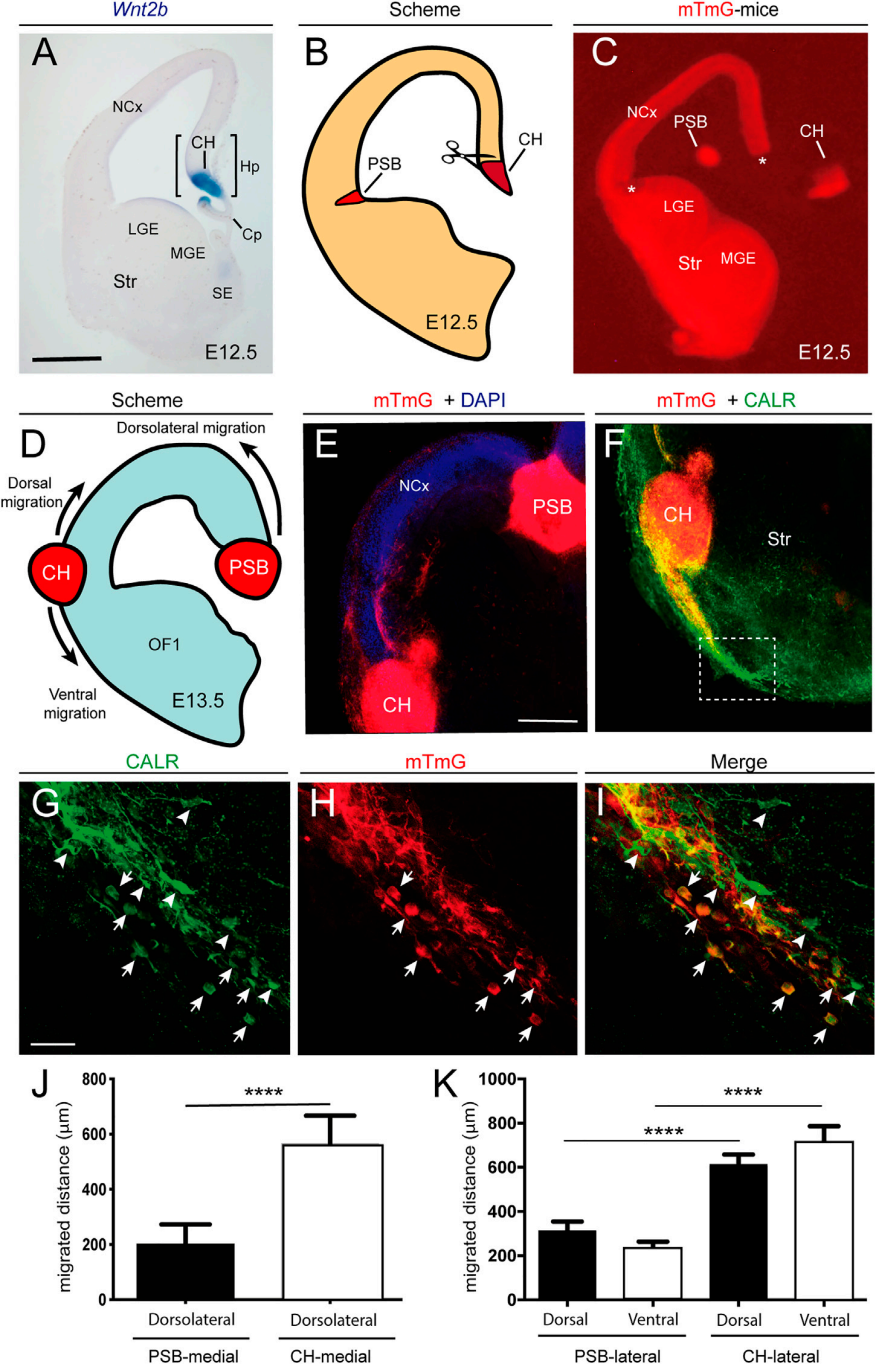
Examples of the differential behavior of CH- and PSB-derived CR cells after transplantation experiments in telencephalic slices **(A)** Example of an E12.5 coronal section showing the location of the CH after *Wnt2b in situ* hybridization **(B,C)** Scheme **(B)** and low-power fluorescence photomicrographs **(C)** illustrating the microdissection procedure for the CH and the PSB using the reporter mTmG mice **(D,E)** Scheme **(D)** and confocal microcopy photomicrographs **(E)** illustrating the location of the transplanted CH and PSB in telencephalic slices **(F)** Photomicrographs illustrating the migratory stream of CH-derived CR cells after their lateral transplantation. The dashed labelled box is depicted in panels G-I **(G,I)** Representative images of double-labelled CR cells identified with CALR antibodies (left) and mTmG (center), together with the resulting combined image (right). White arrows mark cells that are both CALR- and mTmG-positive, while arrowheads point to CALR-positive cells. The panels highlight the large number of double-labelled cells that migrate ventrally to the transplanted CH, that contrasts with the fewer CALR-positive cells **(J)** Bar plots of the migration distance of double-labelled (CALR-mTmG) CR cells in transplantation experiments, comparing the extent of migration of PSB and CH-derived cells transplanted in the natural CH location **(K)** Bar plots of migration distance of double-labelled cells in the dorsal and ventral regions of the CH and PSB transplants after a lateral transplantation. Data on panels **(J,K)** are presented as mean ± s.e.m.. ****p < 0.0001. Abbreviations: Cp = choroid plexus; CH = cortical hem; Hp = hippocampal primordia; LGE and MGE = Lateral and medial ganglionic eminences; NCx = neocortex; SE = septal region; Str = striatum; PSB = pallial subpallial boundary. Scale bars, A = 300 μm pertains to C; E = 200 μm pertains to F and G = 50 μm pertains to **(F–I)**.

Due to the above illustrated data, we aimed to develop ectopic transplantation experiments in an *in vitro* preparation of telencephalic slices (see Materials and Methods for details) (Figure 4). Thus, coronal embryonic telencephalic slices (E12.5) from wild-type mice were cultured on transwells, essentially as described (Bribian et al., 2014), and the endogenous CH and PSB were removed, while the CH and PSB from mTmG reporter mice were transplanted (Figure 4D). Migrated CR cells generated after explant transplantation could be easily identified by their red fluorescence protein (tdTomato) expression, but also by using double labeling with CALR antibodies. Results demonstrated that CH-derived CR cells transplanted in their original position are able to migrate long distances tangentially in dorsal and medial marginal zones of the slices (mTmG CH in CH location = 565.7 ± 101.3 μm, *n* = 9, mean ± s.e.m) (Supplementary Figure S2). However, PSB-derived CR cells were unable to migrate longer distances in dorsal portions of the pallium (mTmG PSB in CH location = 200.7 ± 72.7 μm, *n* = 12; mean ± s.e.m.) (Figures 4D–E). In contrast, when mTmG CH explants were transplanted in the PSB location, a large number of double-labeled CR cells (mTmG + CALR) could migrate dorsally as well as towards ventral portions (mTmG CH in PSB location, lateral-dorsal migration = 603.1 ± 55.0 μm; lateral-ventral migration = 706.7 ± 79.5 μm; *n* = 17; mean ± s.e.m.) (Figure 4F–K). In parallel, mTmG PSB transplanted in the PSB location showed increased migration when compared after transplantation in the CH (mTmG PSB in PSB, dorsal migration = 310.5 ± 44.25 μm; ventral migration = 232.9 ± 30.94 μm; *n* = 11; mean ± s.e.m.) (Figures 4J–K). From these experiments we may conclude that PSB dorsal migration is ≈ 1.55 times greater when transplanted in PSB than in CH regions. In contrast, CH showed ≈ 1.25 times greater migration distances when transplanted in the PSB than in the CH. These data agree with previous TFM results indicating that CR cells originating from the CH were able to generate stronger mechanical forces to the substrate and they migrate in hydrogels ranking from 5.8 mg/ml to 9.8 mg/ml greater than PSB-derived CR cells. Thus, CH-derived CR cells, when transplanted in PSB, were able to strongly migrate both due to the lesser stiffness of the region (as compared to dorsal regions) and to their intrinsic mechanical properties. In contrast, PSB-derived CR cells migrate less towards or within dorsal pallial regions with increased stiffness. Taken together, the present data suggest that both the differing stiffness in marginal zone/layer I (dorsal *vs*. lateral) of the developing pallium and the intrinsic differences in the motogenic properties of the CR cells depending on their origin play a role in determining their distribution in the developing marginal zone as observed *in vivo*.

## Discussion

Several studies in the literature combines lineage analysis, *in vitro* cultures, and CR cell markers, and have revealed the migratory routes of CR cells in developing pallium have been revealed [see among others (Takiguchi-Hayashi et al., 2004; Bielle et al., 2005; Yoshida et al., 2006; Zhao et al., 2006; Garcia-Moreno et al., 2007; Imayoshi et al., 2008; Ceci et al., 2010; Gu et al., 2011; Villar-Cervino et al., 2013; Barber et al., 2015)]. To summarize these studies, CH-derived CR cells migrate in the marginal zone expanding to dorsal and medial parts of the neocortex with a preponderance in the medial level in the caudal axis [see (Barber and Pierani, 2016) as example], since the more rostral levels are mainly populated by SR-generated CR cells. In contrast, PSB-derived CR cells are more confined to lateral portions of the pallial marginal zone with a decreased presence in the medial and dorsal regions [see (Barber and Pierani, 2016) as example]. However, the limits of their distribution in the marginal zone are no hardly defined and these CR populations overlaps in their distribution.

Several groups have focused their attention on defining chemical factors that mediate the migration of the different sets of CR cells (see Introduction). Concerning migration properties of subsets of CR cells, an elegant study carried out by Barber et al. (2015) *.* demonstrated *in vitro* using whole flattened cortical vesicles that CR cells originating in the CH showed increased migratory speed compared to PSB-derived CR cells. The difference (≈ 18%–20% at E10.5) was also associated with differences in VAMP3 expression (increased in CH-derived CR cells with respect to PSB-derived ones). VAMP3 is involved in endocytosis, a crucial process that modulates membrane dynamics at the leading edge of migrating neurons and axons (Kamiguchi and Yoshihara, 2001; Kawauchi, 2015) as occurs in other cell types (Llanses Martinez and Rainero, 2019). In addition, the authors showed that CH-derived CR cells migrate towards the dorsal pallium while PSB-derived CR cells migrate laterally in the rostro-caudal axis (Barber et al., 2015). Our current data corroborate these results.

Concerning expansion of the CR cells in marginal zones, several hypotheses have been proposed. For example, Villar-Cervino et al. (2013), indicated that the distribution of different CR cells is mediated by a contact repulsion process. These effects were not observed when they analyzed groups of CR cells growing in Matrigel™ (Bribian et al., 2014), nor were they seen in present results. However, Barber et al. (2015) *.* suggested that, after analysis of their experiments and time-lapse results, factors other than contact repulsion might regulate CR cell expansion and trajectories in the developing pallium. Our present results are in line with their study, since we show that mechanical factors might contribute to the distribution of CR cells in the dorsal-lateral axis of the developing marginal zone. As indicated, emerging evidence demonstrates that, in parallel to chemical cues, mechanically-mediated processes play relevant roles in axonal guidance, neuronal migration, neurite growth, and brain development [e.g., (Franze, 2013; Gangatharan et al., 2018; Javier-Torrent et al., 2021)]. Concerning pallial morphogenesis, recently published studies from the Miyata’s lab reported new results. In fact, a study of Nagasaka and Miyata (2021) analyzed the stiffness differences between the ventricular zone and the pallium and subpallial ganglionic eminence. Relevantly, in this study the authors reported greater *E* values in the pallial *vs*. the subpallial ventricle, which could be a factor involved in pallial folding (Nagasaka and Miyata, 2021). Our present results reinforce and expand these observations, demonstrating a significant difference for *E* values between the dorsal part of the marginal zone and the lateral regions of the pallium. However, we cannot rule out the possibility that these differences are also linked to those observed in the ventricular zone. From a developmental point of view, during the early stages of cortical development, the pallium expands in thickness with the addition of postmitotic neurons, generating the cortical plate in a lateral-to-medial gradient, but it also expands laterally [see (Bayer and Altman, 1991)]. Concerning the lateral-to-ventral expansion of the neocortex, a study by Saito et al. (2019)*.* described how a migratory stream of the early generated (E10.5) dorsally preplate cells (mainly subplate cells) participated strongly in this lateral pallial expansion, as evidenced by a dorsal-to-lateral migration, and most probably acting on the orientation of radial glial cells and generating axon tensions at the level of subpallium, at E14.5 (Misson et al., 1988). The presence of these subplate corticofugal axons (labelled with anti-GABA antibodies or 1,1′-Dioctadecyl-3,3,3′,3′-Tetramethylindocarbocyanine Perchlorate (DiI) tracing) at E14.5 was also reported by a number of pioneering studies (De Carlos and O'Leary, 1992; Del Rio et al., 1992; Del Rio et al., 2000). Under this scenario, the lateral part of the pallium which is closest to the ganglionic eminence will allow the tangential migration of these preplate-derived cells (Saito et al., 2019). However, whether these early generated VZ-derived subplate cells with monopolar morphology also show mechanical differences with the non-monopolar cells located in the medial-dorsal portions of the subplate warrants further study and is of interest in fitting their functions and behavior into the stiffness differences observed in the developing pallium.

With respect to CR cells, these studies of Miyata’s lab did not focus on this cell type. Our results demonstrate that CH-derived CR cells can migrate long distances in the marginal zone in both the lateral and the dorsal part of the pallium. Interestingly, they were able to migrate more in the lateral than the dorsal regions in our transplantation experiments. In contrast, PSB-derived CR cells showed reduced migration when transplanted into dorsal regions of the neocortex. This observation is likely related to their migratory properties in the differing stiffness of the dorsal *vs*. lateral parts of the marginal zone (BIO-AFM experiments) as corroborated in our Matrigel™ experiments. Taking this into account, our observations and those of Saito et al. (2019) *.* suggest that the migration of CR cells follows the changes in cortical stiffness generated between E10.5 and E12.5. This is of relevance since in the absence of these coordinated actions, CR cell mechanical properties, dorsal-lateral *E* differences in the ventricular and marginal zones, as well as the described dorsal-lateral stream of preplate-derived cells, might trigger altered neocortical development. In fact, it is widely recognized that correct CR cell distribution in marginal zone-layer I is a crucial factor in both radial glia maintenance (Super et al., 2000) and neuronal radial migration (Rice and Curran, 2001). With altered CR cell distribution, changes in cortical plate development and layer specification might occur [e.g., (Super et al., 2000; Alcantara et al., 2006; Villar-Cervino et al., 2013)]. Our study describes for the first time how subsets of CR cells can also be characterized by their mechanical properties which, along with the differential dorsal-lateral stiffness of the marginal zone and additional chemical cues, allow the orchestrated dorsal-lateral migration of two different CR-cell populations (CH and PSB-derived) leading to a specific regional distribution that plays a role in cortical development and maturation.

### Coordinated Action of Mechanical and Chemical Cues Modulates the Dorsal-Lateral Migration and Distribution of CH and PSB-Derived CR Cells: A Putative Scenario in Early Neocortical Development

**FIGURE 5:**
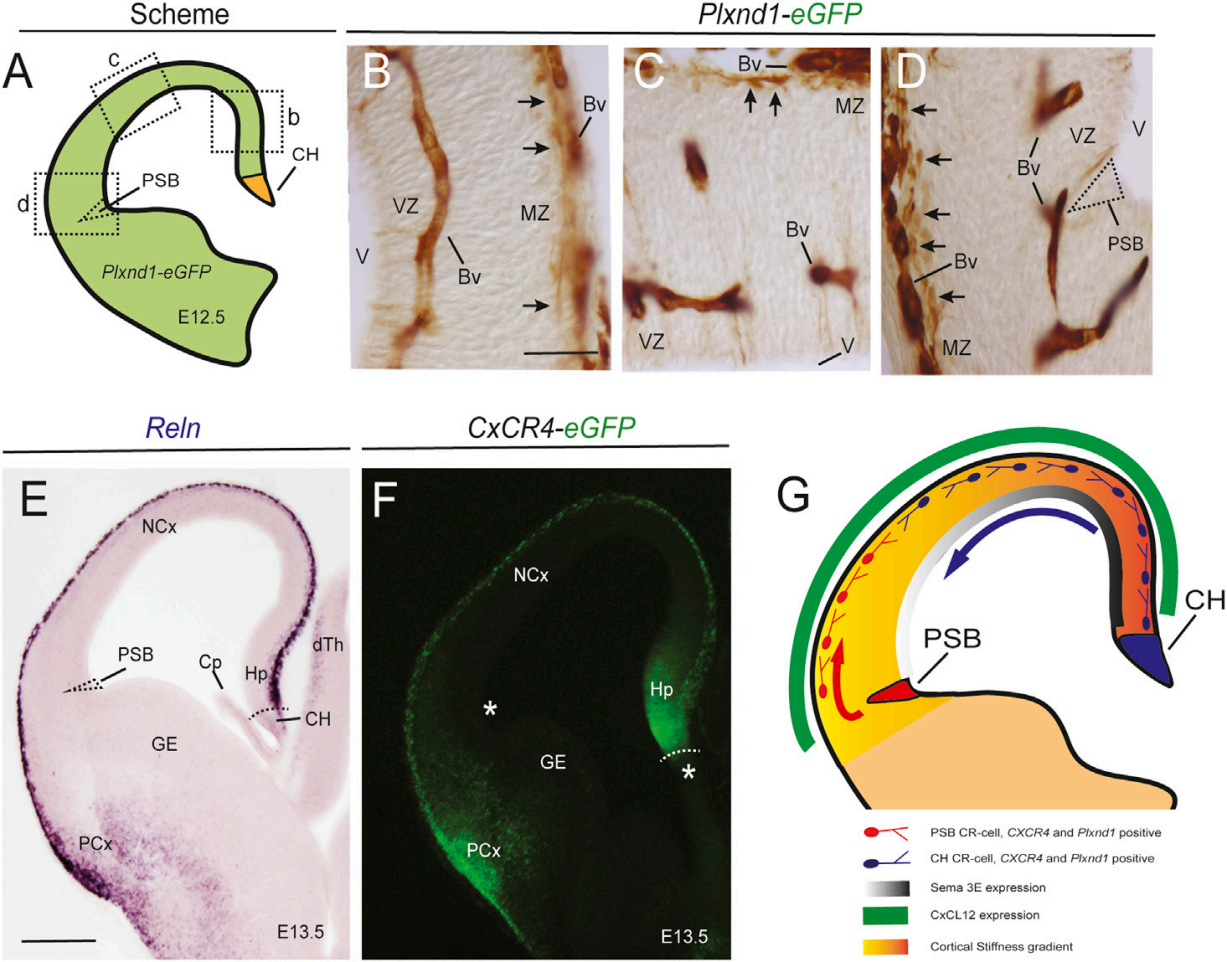
Mechanical and chemical cues modulate the migration of PSB- and CH-derived CR cells. Data presented in this figure illustrate some of the cues involved in the CR-cell migration **(A–D)** Scheme **(A)** and eGFP immunostaining using immunoperoxidase methods of PlexinD1 in transgenic *Plxnd1*-eGFP mice. PlexinD1 is present in blood vessels as well as in CR cells in the complete marginal zone (arrows in B-D). The specific location of the PSB is labelled in **(D)** and illustrates the absence of PlexinD1 in this region. This demonstrates that its expression is postmitotic and linked to its position in marginal zone **(E,F)** *Reln in situ* hybridization **(E)** and eGFP fluorescence in CxCR4-eGFP mice **(F)**, respectively, illustrating their distribution in the developing pallium. Asterisks and dashed regions in **(F)** illustrate the absence of *Reln* and CXCR4 in the proliferative regions, especially the PSB **(G)** Scheme summarizing the results observed in our studies (see Discussion for details). Abbreviations as in Figure 4 in addition to Bv = blood vessel; dTh = dorsal thalamus; GE = ganglionic eminence; MZ = marginal zone; PCx = piriform cortex; V = lateral ventricle; VZ = ventricular zone. Scale bars, B = 50 μm pertains to **(C,D)**; E = 300 μm pertains to **(F)**.

In Figure 5 we hypothesize a putative scenario for the dorsal-lateral CR-cell migration generated in the CH and the PSB. This summary includes results published by several research groups [among others (Bielle et al., 2005; Borrell and Marin, 2006; Bribian et al., 2014; Barber and Pierani, 2016)] and the present results. In this scheme, the dorsal-medial difference in pallial stiffness is illustrated (present results) and the presence of CXCL12 and Sema3E is illustrated. In addition, we include data related to the expression of CXCR4 as well as PlexinD1. In this hypothesis, during development, SR-, CH-, and PSB-derived CR cells are generated in parallel. However, neither the CH nor the PSB express PlexinD1 or CXCR4 (Figures 5A–D,F). In contrast, in the marginal zone, both CH- and PSB-derived CR cells express PlexinD1 and CXCR4, as well as Reelin (Figures 5A–F). Due to the differing stiffness of the pallium, PSB-derived CR cells can migrate in their lateral portions to the marginal zone, while being blocked in dorsal pallial regions displaying greater stiffness and increased Sema3E expression. In contrast, CH cells with greater mechanical properties can migrate in dorsal portions but are also progressively affected by the action of Sema3E (Figure 5G). Both CR-cell populations are positioned in the marginal zone by the action of CXCL12, and their final distribution in the dorsal-lateral axis is promoted by the inhibition of CXCL12/CXCR4 signaling by Sema3E, as demonstrated in (Bribian et al., 2014), along with their differences in intrinsic mechanical properties and the stiffness of the developing pallium (present results). In addition to this, other factors such as the expression of VAMP3 leading to intrinsic differences in CR-cell migration as well as other repulsive actions described in other studies play crucial roles in parallel to accomplish their regional distribution in the rostral-caudal and medial-lateral axis of the pallium (see Introduction for details).

## Data Availability

The original contributions presented in the study are included in the article/Supplementary Material, further inquiries can be directed to the corresponding author.
