# Supplementary material for: Involvement of Mechanical Cues in the Migration of Cajal-Retzius Cells in the Marginal Zone During Neocortical Development
Source: Front Cell Dev Biol. 2022 May 16;10:886110. doi: 10.3389/fcell.2022.886110 (PMC9150848; doi:10.3389/fcell.2022.886110)
Supplement: Supplementary file 2 [file DataSheet1.PDF]

Supplementary Material

Supplementary Material

1 Supplementary Figures and Movies

1.1 Supplementary Figures

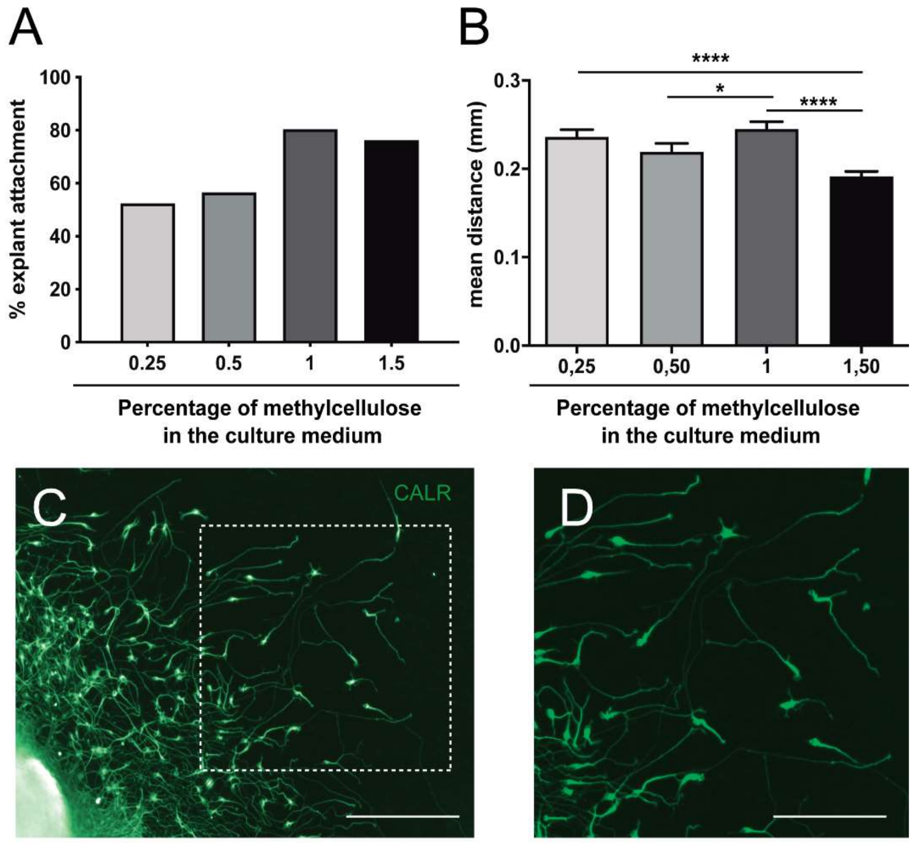

Supplementary Figure 1. Characterization of the explant-derived CR cells growing in 2D cultures with different percentages of methylcellulose. **(A-B)** Histograms illustrating the percentage of explant attachment (A) and CR cells' migrated distance (B) with a difference in the percentage of methylcellulose in the culture media. **(C-D)** Low and high magnification of CR cells growing in 1%

methylcellulose medium after CALR immunostaining showing their monopolar morphology. Data are presented as mean ± s.e.m. **** $p < 0.0001$. Scale bars in C = 150 µm and D = 75 µm.

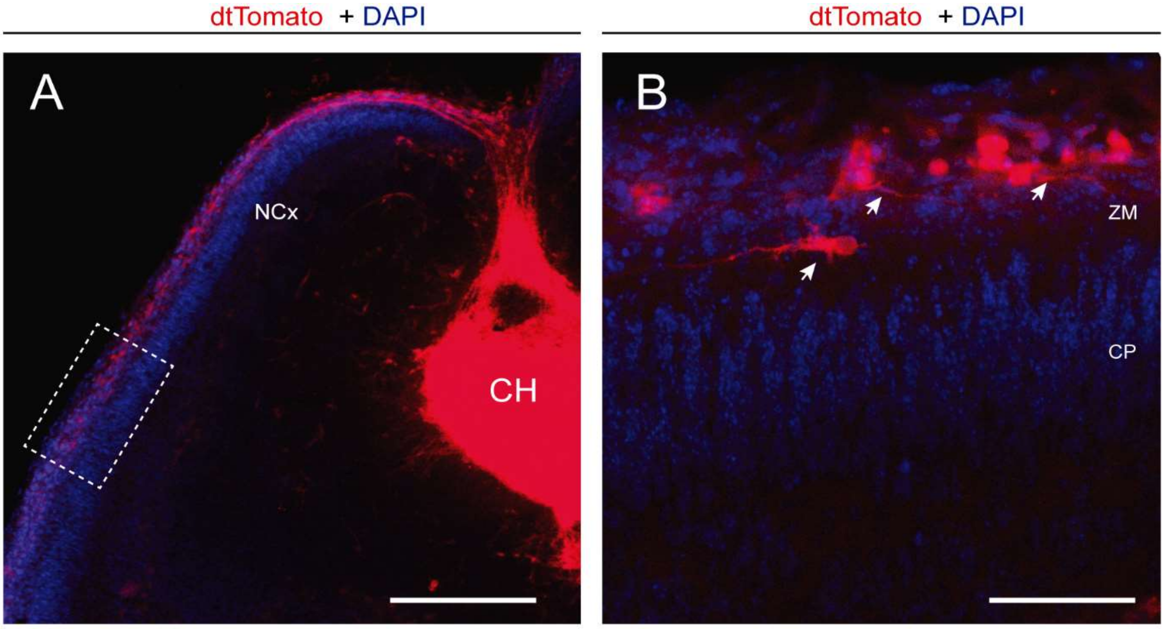

Supplementary Figure 2. mTmG-positive CH-derived CR cells migration in the medial wall of telencephalic slice. **(A-B)** Confocal microscopy images illustrating the migration of the mTmG-positive CR cells after CH explant transplantation in the original location (see Material and Methods for details) of E13.5 OF1 slices. Note the great migration distance of mTmG-positive cells (arrows in (B)) in the dorsal and lateral parts of the marginal zone (MZ) of the cultured OF1 slice. Scale bars in A = 200 µm and B = 50 µm.

1.2 Supplementary Movies

Supplementary 1-2 Movies. Examples of the differential migration between CH and PSB-derived CR cells in different concentrations of Matrigel™ (7.8 and 9.8 mg/ml). Movie frame rate 25 fps. Each frame was obtained after 8 minutes in culture.

Supplementary 3 Movie. Example of the intracellular Ca^{2+} changes in CR cells derived from the CH using Fluo4-AM. For review purposes the movie frame rate was modified at 100 fps. A frame was obtained every 100 ms. A lockup scale (physics) was applied in Fiji™ ranking from blue (0 grey level) up to red (255 grey level). With the present frame rate, in the movie GsTMx-4 was applied at 2 sec and the KCl at 17 sec of the movie. Selected frames of this movie are included in Figure 2.
